# Oral Administration of Freeze‐Dried *Lactobacillus zeae* Alleviates Periodontitis by Affecting the Oral and Intestinal Flora

**DOI:** 10.1002/fsn3.71493

**Published:** 2026-02-01

**Authors:** Qimeng Liang, Zixin Kang, Xiaodong Song, Yuanhong Xie, Hongxing Zhang, Junhua Jin

**Affiliations:** ^1^ Food Science and Engineering College, Beijing University of Agriculture Beijing Laboratory of Food Quality and Safety Beijing China; ^2^ Inner Mongolia Mengniu Dairy (Group) Co. Ltd., Key Laboratory of Dairy Quality Digital Intelligence Monitoring Technology State Administration for Market Regulation Hohhot China

**Keywords:** bacteriostatic, flora, freeze‐dried cells, *Lactobacillus*, periodontitis

## Abstract

Due to the high incidence of periodontal diseases in pet cats and dogs, the purpose of this study is to screen bacteria with beneficial oral health effects and assess the effectiveness of its freeze‐dried cell. In this study, we explored the effects of oral probiotics by determining the bacteriostatic ability, percentages of self‐aggregation and co‐aggregation in vitro, and through microbial community analysis, the alleviating effect of freeze‐dried cells on periodontitis and its mechanism of action were evaluated. *Lactobacillus zeae* N165 significantly inhibited *Fusobacterium nucleatum* and *Porphyromonas gingivalis* , and it had high co‐aggregation rates of 96.78% and 88.94% with *F. nucleatum* and *P. gingivalis* , respectively. In vivo, freeze‐dried *L. zeae* N165 cells significantly reduced alveolar bone resorption, TNF‐α, and IL‐6 levels in rats with periodontitis, maintained a healthy oral and intestinal microbial community structure, and regulated the dominant species to alleviate periodontitis. The prediction of functions by the KEGG database analysis of oral flora revealed that freeze‐dried *L. zeae* N165 cells may alleviate periodontitis via four pathways: reduction of glutamine degradation, modulation of lipopolysaccharide levels, biosynthesis of polyketide glycan units, and biotin metabolism. *L. zeae* N165 showed promising results in both in vitro and in vivo experiments, suggesting new directions for the oral probiotics industry.

## Graphical abstract

First, probiotic strain *Lactobacillus zeae N165* with oral antibacterial activity, autoaggregation ability and coaggregation ability was screened through in vitro experiments. Then, in vivo efficacy evaluation was conducted on this strain, and a mouse model was used to verify its ameliorative effect on periodontitis. The results demonstrated that this strain could alleviate alveolar bone resorption, reduce the levels of pro‐inflammatory factors TNF‐α and IL‐6, maintain the structural stability of oral and intestinal microbiota, and regulate the composition of dominant bacterial species. To further explore the potential metabolic and molecular mechanisms underlying the alleviation of periodontitis by *Lactobacillus zeae N165*, KEGG pathway analysis was performed. It was found that this process was mediated by reducing glutamine degradation, regulating lipopolysaccharide levels, biosynthesizing polyketide units, and metabolizing biotin.

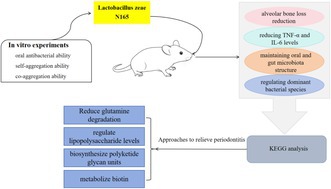

## 1 Introduction

According to veterinary clinical statistics, the incidence of periodontal diseases in pet cats and dogs is approximately 70% and 80%, respectively (Özavci et al. 2019). In addition, the global incidence of adult periodontitis is very high, reaching 80%–90% in China (Jiao et al. 2021). Periodontitis is not only an oral disease; it can cause inflammatory reactions in other organs and nerves (Ray 2023).

The oral cavity is a complex microbiological system, housing an estimated six billion strains of bacteria, with over 700 different species residing in the mouth (Gönczi et al. 2021). The homeostasis of the oral microbiota plays a vital role in maintaining oral health. Disruption of this balance can lead to oral infectious diseases, including periodontitis, dental caries, pulpal and periapical infections, and oral mucositis (Jiang et al. 2021). Oral problems are often associated with infections caused by pathogenic bacteria. Common oral pathogens include *Streptococcus mutans* (Bhaumik et al. 2021), *Candida albicans* (Zhang, Li, et al. 2021; Adam and Khan 2021), *Fusobacterium nucleatum* (Tefiku et al. 2020), and *Porphyromonas gingivalis* (Fiorillo et al. 2019). *F. nucleatum* is one of the causative organisms of bad breath, as it degrades sulfur‐containing amino acids, producing unpleasant volatile sulfides (Liu et al. 2013). *P. gingivalis* and *F. nucleatum* are considered major pathogens associated with periodontal disease (Zhang, Xu, et al. 2021). It is important to maintain a healthy balance of oral microbiota and manage the presence of pathogenic bacteria to prevent oral health problems.

Probiotic therapy is increasingly being utilized as a means to prevent and alleviate oral health issues, particularly in response to the overuse and misuse of antibiotics and the emergence of antibiotic‐resistant strains of bacteria (Fang et al. 2018; Nguyen et al. 2021). Various probiotic bacteria can contribute to oral health by forming barriers through co‐aggregation with pathogenic bacteria, adhering to epithelial cells to prevent the adhesion of pathogenic bacteria and thus limiting their ability to cause infections, competing for nutrients, producing antimicrobial substances, and regulating immune responses (Markowiak and Śliżewska 2017). This plays an important role in the prevention and treatment of oral diseases such as dental caries and periodontal disease. The application of probiotic therapy in oral health aims to restore and maintain a healthy balance of oral microbiota, inhibit the growth of pathogenic bacteria, and promote overall oral health. However, further research is needed to determine the optimal strains and treatment regimens for probiotic therapy.

*Streptococcus salivarius* K12, which was first isolated from the saliva of a healthy child in 1989, has been extensively studied for its probiotic effects on the oral cavity. Numerous experiments have demonstrated its favorable effects (Cernioglo et al. 2021; Wang et al. 2021; Stašková et al. 2021). A study has found that the cell‐free supernatant of *Lactobacillus brevis* strain has antibacterial effect on *Streptococcus mutans* , which can inhibit the cell adhesion of *Streptococcus mutans* and eliminate the biofilm formed by it (Kim et al. 2022). Another study discovered *L. plantarum* K41 isolated from traditional Sichuan pickles effectively inhibited *S. mutans* biofilm formation and thus possesses a potential inhibitory effect on dental caries in vivo (Zhang et al. 2020). The adhesion ability of oral probiotics to oral tissues is a key factor in improving oral health. Probiotics need to colonize the oral environment to maintain a balance between probiotics and pathogenic bacteria. This not only improves the oral microbiota but also exerts a positive impact on oral health by regulating the oral ecological balance disrupted by conditions such as periodontal disease, dental caries, and oral mucositis (Saïz et al. 2021). However, most previous studies have focused on the effects of fresh probiotic cells on oral health. The effectiveness of freeze‐dried cells, the most common application form, remains unknown. Further research is needed to explore the potential benefits and efficacy of freeze‐dried probiotic cells in oral health management and the prevention of oral diseases.

In this study, we first screened bacterial strains with oral probiotic potential through in vitro experiments, including the determination of their oral bacteriostatic ability, self‐aggregation, and co‐aggregation abilities. We then evaluated the prevention and treatment effects of the freeze‐dried cells of the screened strains on oral periodontitis and explored the possible mechanisms through animal experiments.

## 2 Materials and Methods

### 2.1 Laboratory Analysis of Screening Strains

*F. nucleatum* (ATCC 23726) and *P. gingivalis* (ATCC 33277) were conserved in the laboratory of Beijing University Of Agriculture (Beijing, China). *L. zeae* N165 (CGMCC 26771) was cultured in De Man‐Rogosa‐Sharpe (MRS) medium (1 L medium contained 10 g tryptone, 10 g beef paste, 5 g yeast powder, 2 g KH_2_PO_4_, 2 g ammonium citrate, 2 g anhydrous sodium acetate, 20 g dextrose, 1 mL Tween 80, 0.5 g MgSO_4_·7H_2_O, 0.25 g of MnSO_4_‐H_2_O, add 1000 mL of deionized water to dissolve fully, adjust pH to 6.48–6.5) at 37°C for 12 h, *Ss*.K12 was cultured in standard M17 (Solarbio, Beijing, China) medium at 37°C for 12 h. *F. nucleatum* was grown on Trypticase Soy Broth (TSB) medium (Solarbio, Beijing, China) supplemented with yeast dipping powder (5 g/L), cysteine hydrochloride (0.5 g/L), and maintained in an anaerobic environment at 37°C for 12 h. *P. gingivalis* was grown on TSB medium (Solarbio, Beijing, China) supplemented with yeast dipping powder (5 g/L), cysteine hydrochloride (0.5 g/L), vitamin K1 (0.5 μg/mL, Hopebio, Qingdao, China) and 0.5% hemin (1 μg/mL, Macklin, Shanghai, China) and maintained in an anaerobic environment at 37°C for 3 days. When *P. gingivalis* was cultured on TSB agar, 5% Sterile Defibrillated Sheep's Blood (Solarbio, Beijing, China) should be added.

The bacteriostatic activity of the strains was determined by Oxford cup method. The activated second generation strain was inoculated into MRS medium at 2%, incubated at 37°C for 24 h, centrifuged at 4°C and 10,000 r/min for 10 min, and the supernatant was retained and filtered through 0.22 μm filter membrane to remove bacteria. *F. nucleatum* and *P. gingivalis* were used as indicator bacteria, and the inoculum concentration of the indicator bacteria was 10^6^ CFU/mL, and the volume of the indicator bacteria was 100 μL to make the indicator plate. 100 μL of cell‐free supernatant was aspirated into an Oxford cup, and MRS liquid medium was used as a negative control. 4°C for 2 h, and then incubated at 37°C until the appropriate time, and the size of the ring of inhibition was measured by vernier calipers and recorded.

Auto‐aggregating capacity of potential strains was measured as follows: Preparation of bacterial suspension of alternative strains: referring to the method in previous study (Kim et al. 2022) with slight modification, after 24 h of culture of strains, centrifugation was performed at 4°C, 10,000 r/min for 10 min, the bacterial cells were collected, and the bacterial cells were washed twice by PBS, then the bacterial cells were suspended in PBS, and the absorbance was adjusted at a wavelength of 600 nm to be (0.6 ± 0.02), which was recorded as A_0_. Referring to the research method of Piwat et al. (2015), 4 mL of bacterial suspension of the alternative strains was vortexed for 10 s and the absorbance value at OD_600_ was determined after incubation for 1 and 24 h at 37°C. Auto‐aggregation rate (%) = (*A*_0_ − *A*_*t*_)/*A*_0_ × 100. *A*_0_—Initial absorbance value of bacterial suspension, *A*_*t*_—Absorbance value of the suspension of the bacterial suspension after incubation.

Co‐aggregating capacity of potential strains was measured as follows: Referring to the research method of Piwat et al. (2015), alternative strains of bacterial suspensions and pathogenic bacterial suspensions were mixed in equal proportions, and the absorbance values at OD_600_ were measured after incubation at 37°C for 1 and 24 h. Co‐aggregation rate (%) = [(*A*_*x*_ + *A*_*y*_) − 2*A*_*t*(*x*+*y*)_]/(*A*_*x*_ + *A*_*y*_) × 100. *A*_*x*_—Initial absorbance values of pathogenic bacterial suspensions, *A*_*y*_—Initial absorbance value of alternative strain suspension, *A*_*t*(*x*+*y*)_—Absorbance values of the supernatant of the mixed bacterial suspension after standing incubation.

### 2.2 Study Design

48 male wistar rats of 6–8 weeks old of SPF grade, purchased from Beijing Vital River Laboratory Animal Technology Co. Ltd. and housed in the clean laboratory animal room of Beijing University Of Agricultural at a temperature of 22°C ± 2°C, humidity of 50% ± 10%, and 12 h/12 h day/night. All operations were performed in accordance with the regulations of the Animal Ethics Committee of Beijing University Of Agricultural.

The rats were randomly divided into five groups according to the principle of no significant difference in the average body weight of each group, namely the blank control group (CK), the model group (M_PD), the *L.casei* 77 live group (L_77), the *L.zeae* N165 live group (L_N165), and the *S.salivarius* K12 live group (L_K12). Among them, *S.salivarius* K12 is a commercial strain of oral probiotics that prevents immune activation induced by periodontal disease pathogens (MacDonald et al. 2021), so it was set as a positive control strain.

### 2.3 Treatments and Feeding

**FIGURE 1:**
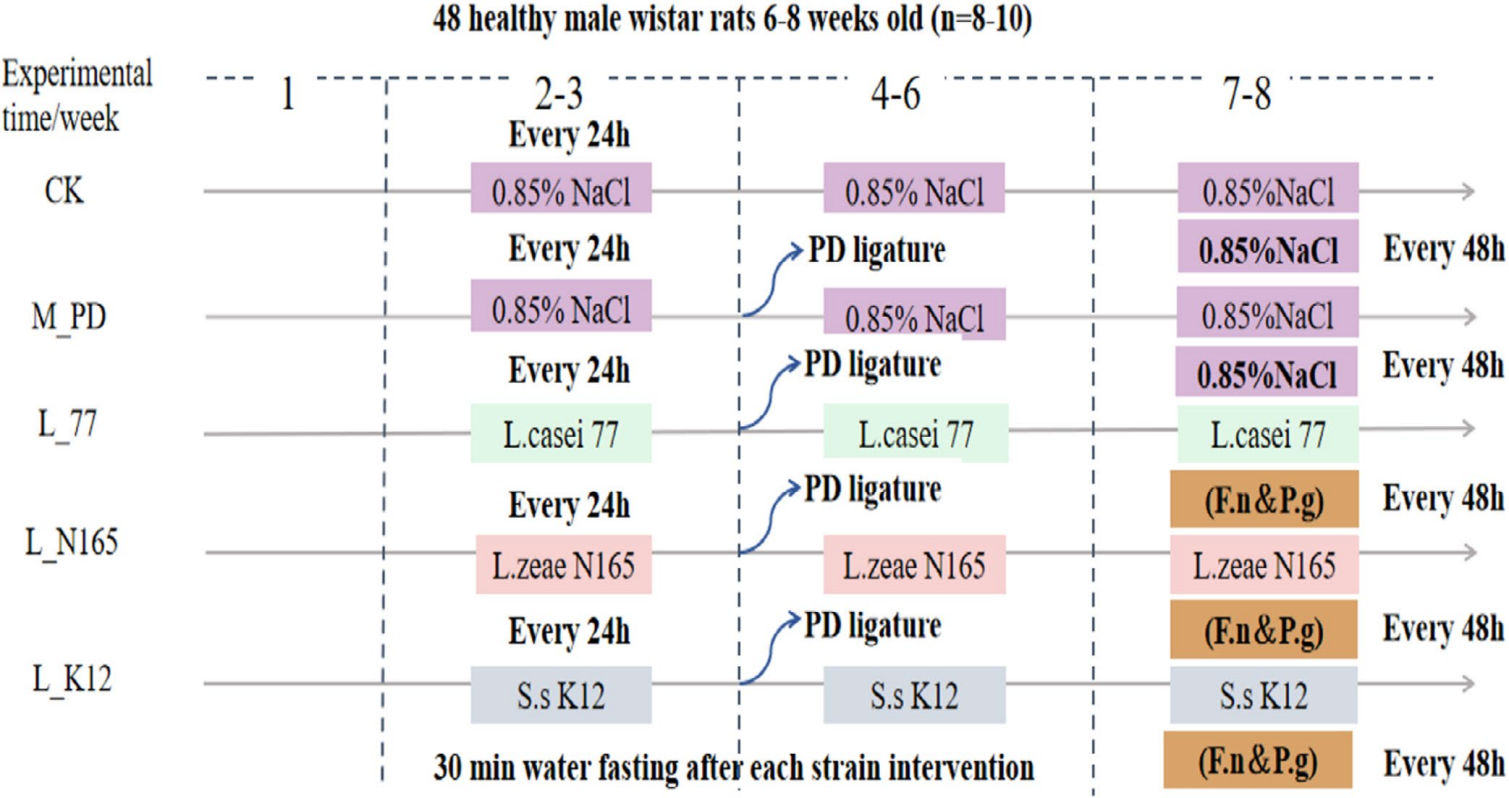
Animal experimental procedures.

The treatment and feeding method of animals experiment was shown in Figure 1. In the 1st week of the experiment, rats were acclimatized and fed ad libitum with food and water. Weeks 2–3 were the pre‐intervention phase of lactobacillus: rats in group 77, N165, and K12 were given 600 μL/kg/day of the corresponding bacterial suspension (10^9^ CFU/mL), and rats in group CK and the model group were given 600 μL/kg/day of sterile 0.85% saline. The intervention was performed by slowly drenching the whole oral cavity of the rats with the suspension or saline using a disposable sterile syringe needle and swallowing it into the stomach, and water was forbidden for 30 min. Weeks 4–6, were the simultaneous phase of modeling and lactobacillus intervention by silk ligation method for periodontitis. Except for the CK group, rats in other groups were anesthetized and silk ligated with a sterile 3–0 non‐absorbable surgical wire on the bilateral maxillary second molars, with the buccal side knotted (Wu, Fang, et al. 2022; Wu, Han, et al. 2022). The ligated wires were observed for 3 days/times and promptly re‐ligated when detachment was detected. *Lactobacillus* maintenance intervention, method and frequency were the same as Weeks 2–3. Weeks 7–8, a simultaneous phase of periodontitis silk ligation method with periodontal pathogen infestation combined with modeling and lactobacillus intervention. In this experiment, at the 7th week of the experiment, when the ligature wire was stable and the ligature wound recovered naturally, *F. nucleatum* and *P. gingivalis* were infested. A mixture of *F. nucleatum* and *P. gingivalis* suspensions (10^9^ CFU/mL) of 600 μL/kg/dose was given to rats in the model group, group 77, group N165, and group K12 at a frequency of 1 time/48 h (Zhang, Xu, et al. 2021), and 0.85% saline was used as a substitute in the CK group. The infestation was carried out in the same way as the lactobacillus intervention, while the lactobacillus maintained the intervention with the same method and frequency as in Weeks 2–3.

### 2.4 Recording and Sampling

At the end of the experiment in the 8th week, rats were anesthetized for oral sampling using an oral sampling swab, and blood was taken from the abdominal aorta, centrifuged at 4°C for 10 min at 4000 × *g*, and the upper layer of clear liquid was collected as serum, which was frozen and stored at −80°C for spare use. The gingival tissues, maxilla, and rectal contents of rats were rapidly removed. The rat maxilla was fixed with 4% neutral formaldehyde solution and stored at room temperature for Micro‐CT analysis; oral swabs and the rest of the tissues were rapidly frozen and stored in liquid nitrogen. Gingival tissues were used for ELISA analysis, and oral swabs and rectal contents were used for flora diversity analysis.

### 2.5 Laboratory Analysis of Animal Experiment

In this study, X‐ray scanning imaging of rat maxillary samples was performed by Beijing Xin Aikemei Technology Co. Ltd. to determine alveolar bone level (ABL): the distance from the enamel bone boundary of the second molar to the top of the alveolar ridge was measured at four sites per tooth: buccal, proximal, and distal on the lingual side. The average of the measurements at each site is the amount of alveolar bone resorption for that tooth. IL‐6 and TNF‐α indexes were measured by ELISA on rat gingival tissues by Beijing Jinglai Huake Biotechnology Co. The microbiological diversity of rat oral swabs and intestinal contents was examined by Shanghai Meiji Biomedical Technology Co. Ltd. Total microbial genomic DNA was extracted from rat's oral and feces samples using the FastPure Stool DNA Isolation Kit (MJYH, shanghai, China) according to manufacturer's instructions. The quality and concentration of DNA were determined by 1.0% agarose gel electrophoresis and a NanoDrop ND‐2000 spectrophotometer (Thermo Scientific Inc., USA) and kept at −80°C prior to further use. The hypervariable region V3‐V4 of the bacterial 16S rRNA gene was amplified with primer pairs 338F (5′‐ACTCCTACGGGAGGCAGCAG‐3′) and 806R (5′‐GGACTACHVGGGTWTCTAAT‐3′) by a T100 Thermal Cycler (BIO‐RAD, USA). The PCR reaction mixture included 4 μL 5 × Fast Pfu buffer, 2 μL 2.5 mM dNTPs, 0.8 μL each primer (5 μM), 0.4 μL Fast Pfu polymerase, 10 ng of template DNA, and ddH_2_O to a final volume of 20 μL. PCR amplification cycling conditions were as follows: initial denaturation at 95°C for 3 min, followed by 29 cycles of denaturing at 95°C for 30 s, annealing at 53°C for 30 s, and extension at 72°C for 45 s, and single extension at 72°C for 10 min, and end at 4°C. All samples were amplified in triplicate. The PCR product was extracted from 2% agarose gel and purified. Then quantified using Synergy HTX (Biotek, USA).

### 2.6 Statistics and Analysis

The experimental results were statistically analyzed using SPSS software with version 20.0, and Duncan analysis was used when the variance between groups was homogeneous, and Dunnett T3 analysis was used when the variance was not homogeneous, in which *p* < 0.05 was used to indicate that the data between groups were significantly different. The microbial diversity test results were analyzed in the Meiji cloud platform for raw confidence.

## 3 Results

### 3.1 Measurement Results for Oral Bacteriostatic Capacity，Auto‐Aggregation and Co‐Aggregation Abilities

**FIGURE 2:**
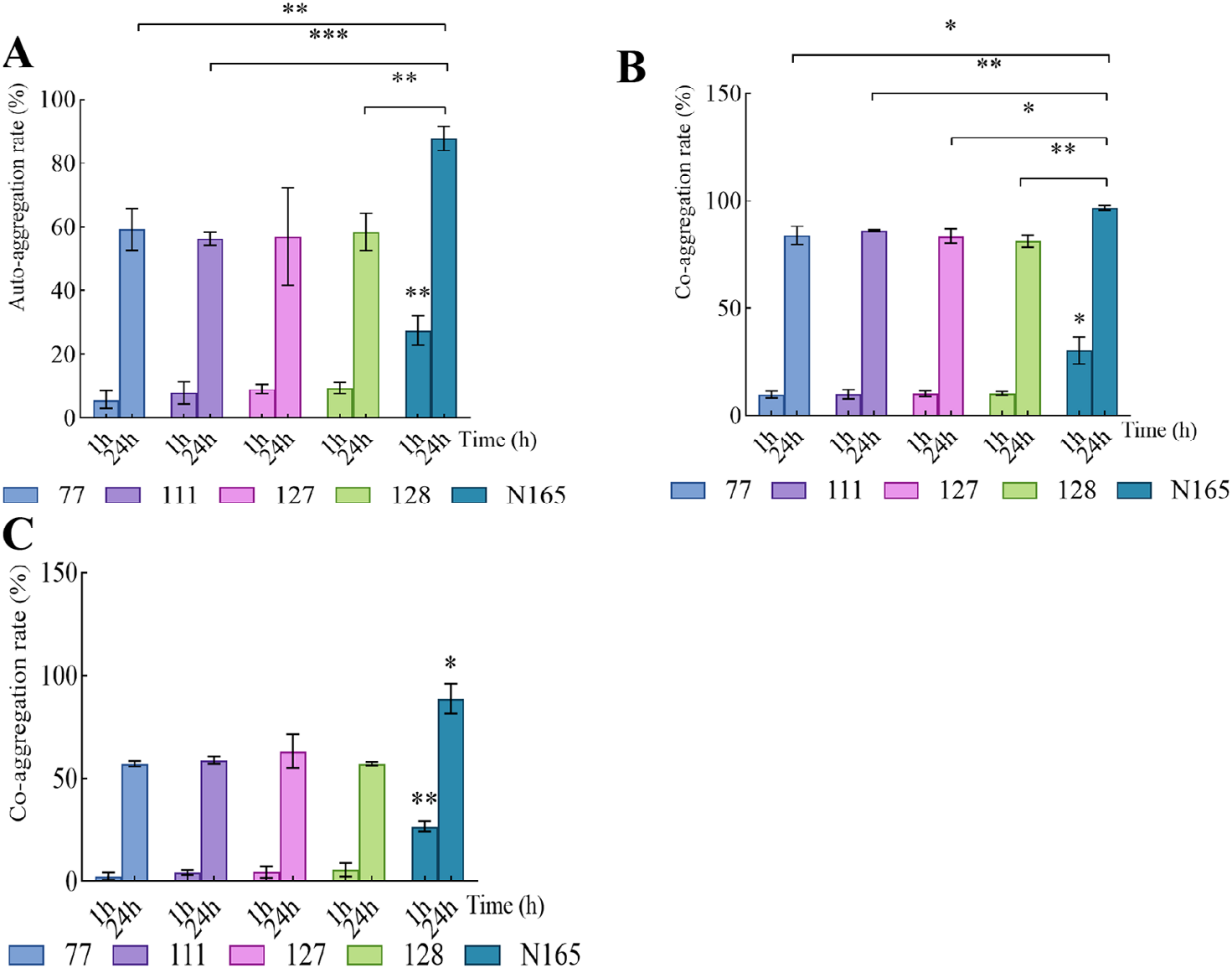
Bacteriostasis and aggregation ability of different strains (A) Auto‐aggregation rate, (B) co‐aggregation rate with *F. nucleatum* , (C) co‐aggregation rate with *P. gingivalis* . The data are all represented by mean with SD. The significance mark indicates: *p < 0.05, **p < 0.01, ***p < 0.001.

The results for the inhibitory effect on *F. nucleatum* and *P. gingivalis* of potential strains showed that *L. casei* 77, *L. casei* 111, *L. paracasei* 127, *L. paracasei* 128, and *L. zeae* N165 presented good antibacterial capability (Table 1). Figure 2 shows the self‐aggregation and co‐aggregation rates of different strains at 1 and 24 h. The self‐aggregation abilities of the five *Lactobacillus* strains and the co‐aggregation ability of *F. nucleatum* and *P. gingivalis* were positively correlated with time, demonstrating their temporal dependence. Notably, the self‐aggregation and co‐aggregation rates of *L. zeae* N165 were significantly different from those of the other four strains (*p* < 0.05), with a self‐aggregation rate of 87.75% and co‐aggregation rates with *F. nucleatum* and *P. gingivalis* as high as 96.78% and 88.94%, respectively, at 24 h.

**TABLE 1:** Antibacterial effect of oral bacteriostatic potential strains.

Potential strain	Size of inhibition circle/mm		Source
	*G. nucleatum*	*P. gingivalis*	
*L. casei* 111	21.38 ± 1.57	14.74 ± 0.97	This study
*L. casei* 77	20.93 ± 3.19	11.40 ± 4.96	This study
*L. paracasei* 127	20.71 ± 3.00	11.31 ± 2.92	This study
*L. paracasei* 128	20.64 ± 1.55	12.33 ± 3.55	This study
*L. zeae* N165	18.41 ± 1.60	12.58 ± 2.93	This study

### 3.2 Micro‐CT Analysis of Rat the Maxilla Bone TNF‐α and IL‐6 Levels in Rat Gingival Tissues

**FIGURE 3:**
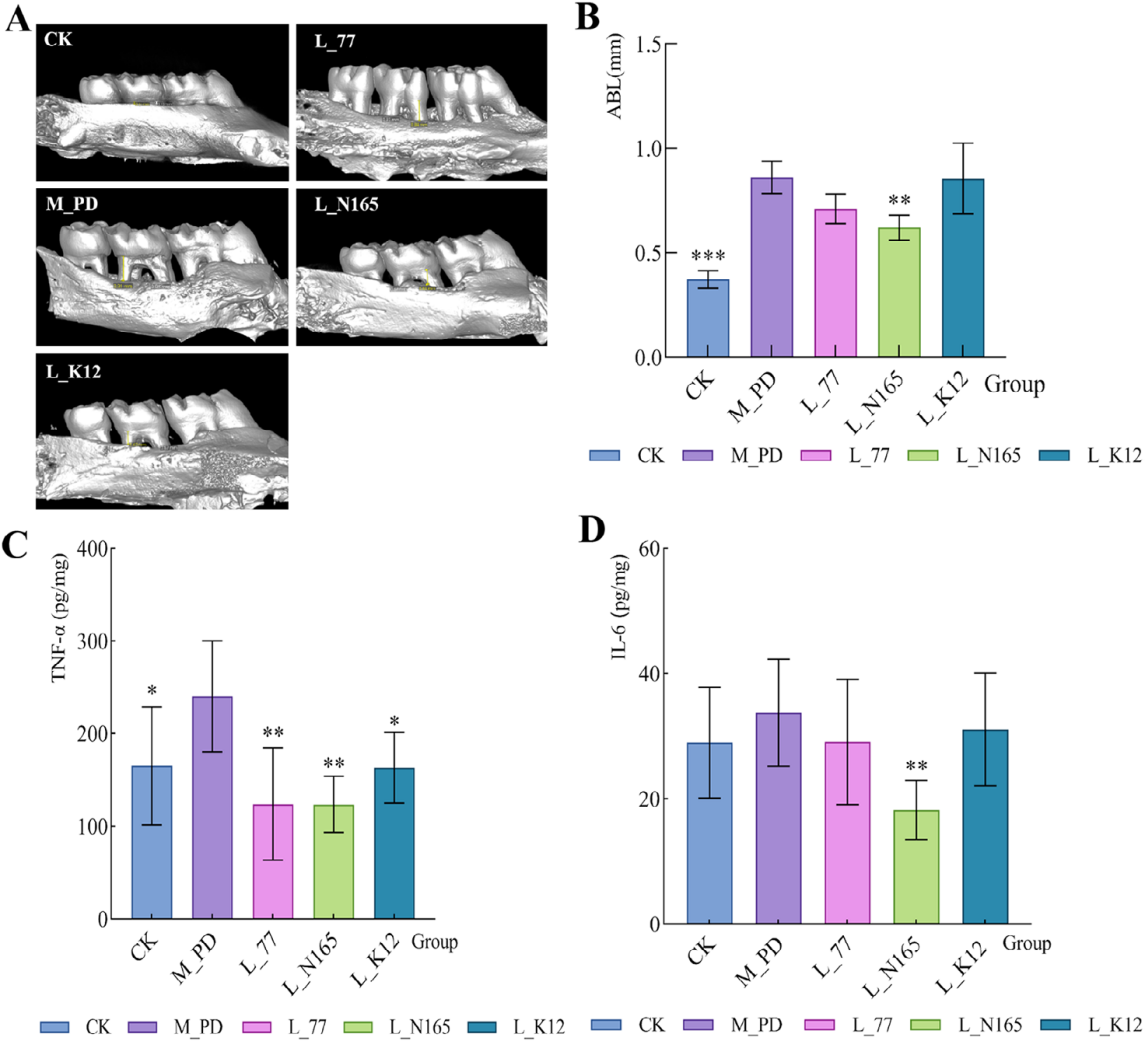
Micro‐CT and levels of inflammatory factors in gingival tissue in rats (A) Micro‐CT maps of rats in each group, (B) ABL, (C) TNF‐α, (D) IL‐6. The data are all represented by mean with SD. The significance mark indicates: Other group VS M_PD group, *p < 0.05, **p < 0.01, ***p < 0.001.

The model group had severe alveolar bone loss compared to the CK group, with significant root exposure, apparent root resorption, and a non‐smooth surface. In contrast, the alveolar bone loss in group 77, group N165, and group K12 was less severe, with no apparent root resorption and a smoother surface (Figure 3A). The results of the alveolar bone resorption measurements are shown in Figure 3B. The alveolar bone resorption in the model group was significantly higher than that in the CK group (*p* < 0.05). *L. casei* 77, *L. zeae* N165, and *S. salivarius* K12 reduced alveolar bone resorption caused by periodontitis by 30.76%, 49.25%, and 1.02%, respectively. The alveolar bone resorption was significantly reduced by *L. zeae* N165 (*p* < 0.05). There was no significant difference in the K12 group (*p* > 0.05), similar to previous findings (Wu, Fang, et al. 2022; Wu, Han, et al. 2022).

In this study, the concentrations of inflammatory factors in rat gingival tissues were determined. There was a significant difference in TNF‐α between the CK group and the model group (*p* < 0.05), and the intake of *L. casei* 77, *L. zeae* N165, and *S. salivarius* K12 significantly reduced the concentration of TNF‐α (*p* < 0.05) (Figure 3C). The IL‐6 levels tended to increase in the model group compared to the CK group, but there was no significant difference (*p* > 0.05) (Figure 3D). *L. casei* 77, *L. zeae* N165, and *S. salivarius* K12 all reduced the concentration of IL‐6, but only the N165 group showed a significant down‐regulation effect.

### 3.3 Effects of Strains on the Oral Flora of Rats

**FIGURE 4:**
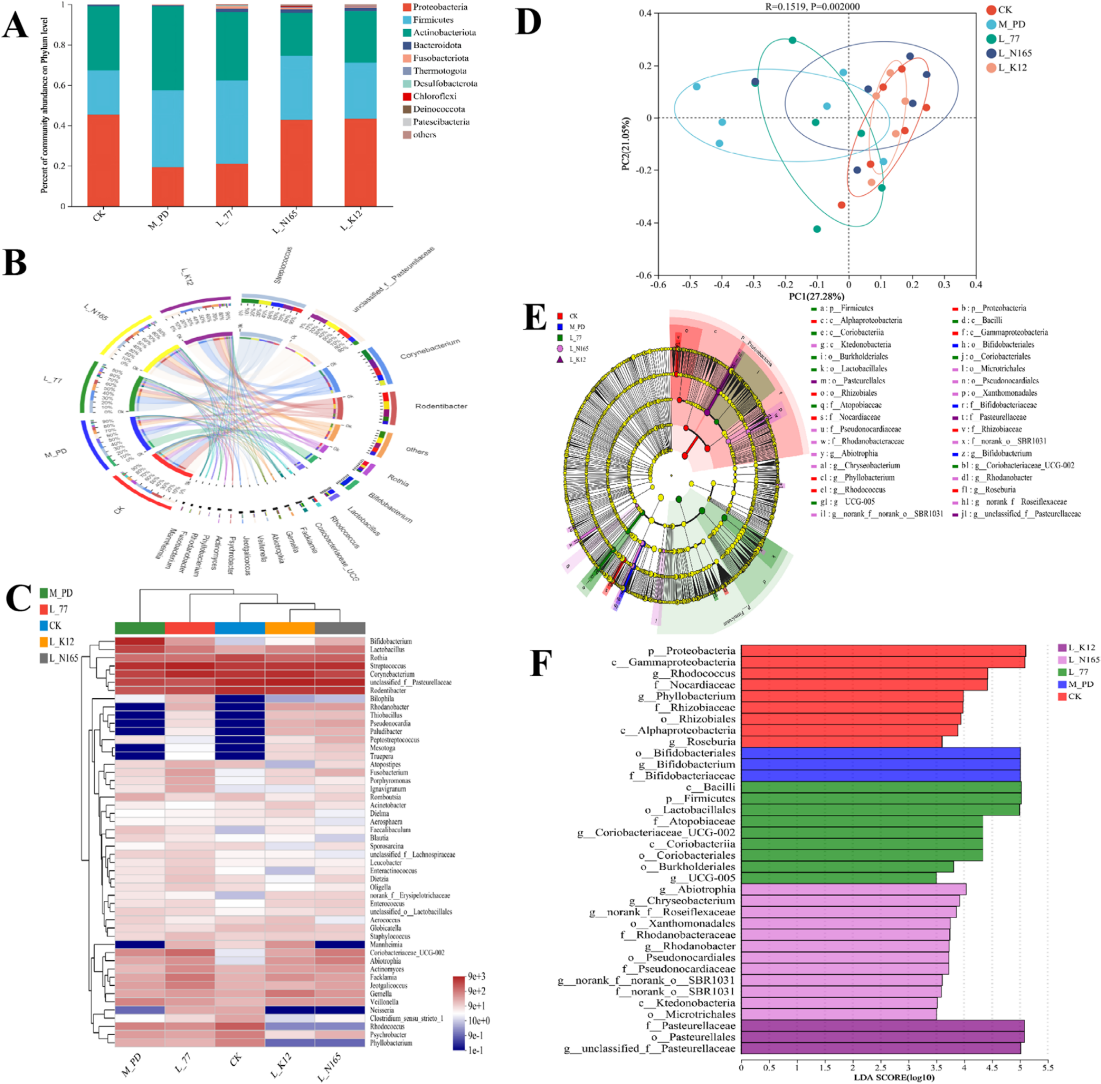
Effects of strain on oral flora in rats. (A) Effects of phylum level, (B) circos plot of species distribution at the genus level, (C) heat map at the genus level, (D) analysis results of PCoA at genus level, (E) LEfSe multilevel species hierarchical tree diagram, and (F) LDA discrimination results. LDA threshold 3.5.

We analyzed the diversity of oral and intestinal flora in rats, and the results were as follows: The tendency of lactobacillus to modulate the oral flora during the alleviation of periodontitis is illustrated in Figure 4A that shows the histogram of the community at the portal level. The three dominant groups of flora in the oral cavity were *Proteobacteria*, *Firmicutes*, and *Actinobacteriota*. The abundance of *Proteobacteria* in the model group was significantly lower than that in the CK group, and the abundance of *Firmicutes* was significantly higher than that in the CK group. Under the intervention of *L. zeae* N165 and *S. salivarius* K12, the abundances of *Proteobacteria* and *Firmicutes* showed significant modulating effects. However, none of the above phenomena were evident in the *L. casei* 77 group. In addition, the abundance of *Actinobacteriota* was higher in the model group than in the CK group, and its abundance was reduced by the intervention of *L. casei* 77, *L. zeae* N165, and *S. salivarius* K12.

Further exploration of the differences between the groups at the genus level is shown in Figure 4B. *Streptococcus*, *unclassified_f_Pasteurellaceae*, *Corynebacterium*, and *Rodentibacter* were the dominant genera of the oral flora. Among these, *Corynebacterium* and *Rothia* are biomarkers of a healthy periodontium (Cai et al. 2021). The abundances of *Corynebacterium*, *Rothia*, and *unclassified_f_Pasteurellaceae* were down‐regulated in the model group compared to the CK group, whereas the abundance of *Corynebacterium* in the *L. casei* 77 group, the abundance of *Rothia* and *unclassified_f_Pasteurellaceae* in the N165 group, and the abundance of *unclassified_f_Pasteurellaceae* in the K12 group were increased. In contrast, the abundance of the model group in *Veillonella* was higher than that of the CK group, being reduced by *L. casei* 77, *L. zeae* N165, and *S. salivarius* K12. A heat map of the community structure at the genus level is shown in Figure 4C, where the oral community structure of the N165 and K12 groups was significantly different from that of the model group and was similar to that of the CK group.

To further investigate the effect of lactobacilli on the floral structure of periodontitis, the present study carried out a β‐diversity analysis at the genus level of the oral flora and constructed a Bray‐Curtis distance matrix for PCoA analysis. There was a significant difference in the oral flora structure between the model group and the CK group; the *L. zeae* N165 group, the K12 group, and the CK group were similar, and the effect of the *L. casei* 77 group was unclear (Figure 4D). To further determine the dominant species in the relief of periodontitis in each subject group, we conducted a LEfSe multilevel species difference analysis (Figure 4E). The dominant species in group 77 were *c_Bacilli*, *p__Firmicutes*, and *o__Lactobacillales*, and in group N165 the dominant species was *g__Abiotrophia*. In group N165, the dominant species were *g__Abiotrophia* and *g__Chryseobacterium*. In group K12, the dominant species were *f__Pasteurellaceae* and *o__Pasteurellales*.

**FIGURE 5:**
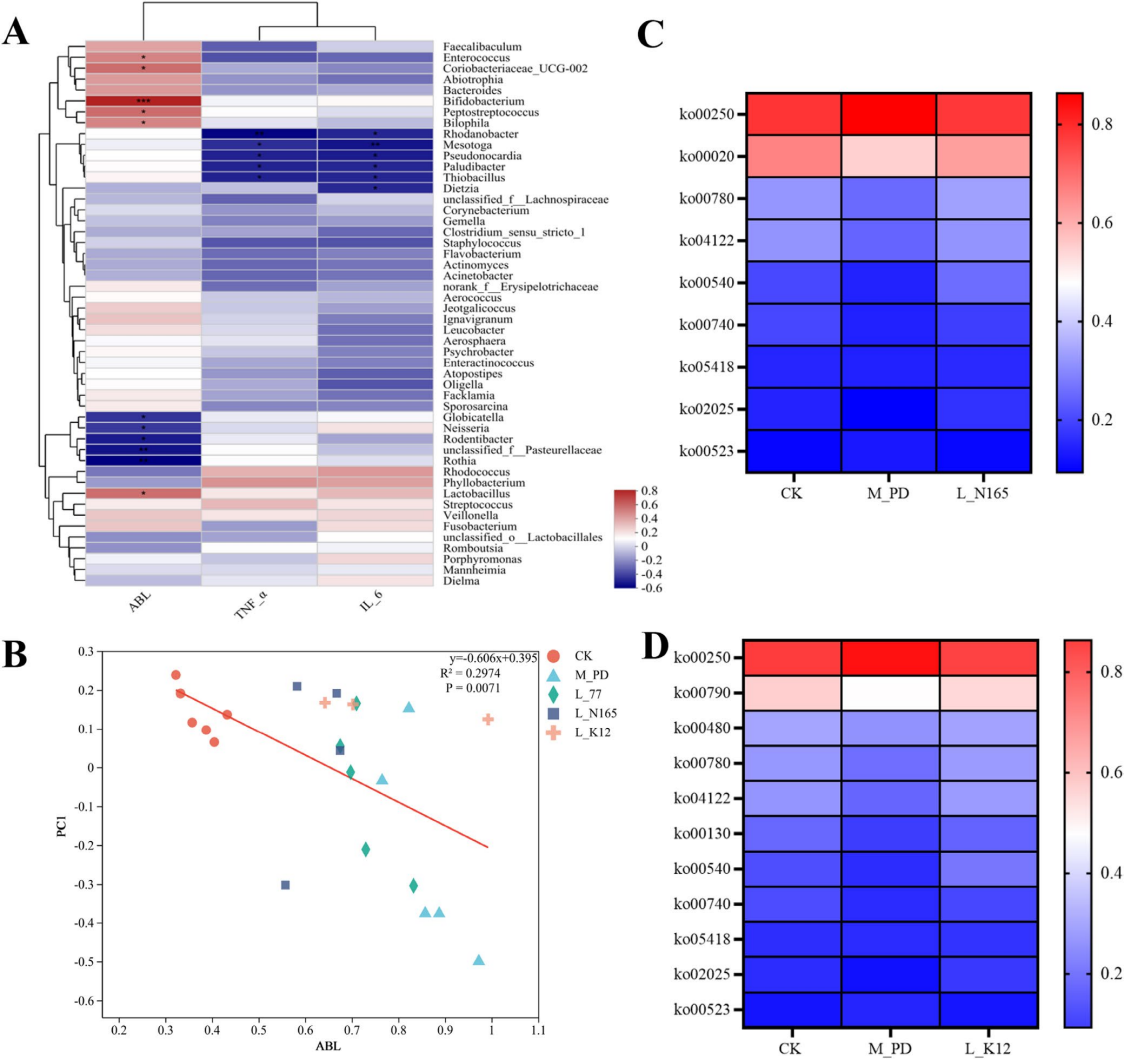
The correlation between oral flora and pathological indexes and the results of enrichment pathway. (A) Spearman's correlation heatmap of TNF‐α, IL‐6, and ABL values with oral flora, (B) plot of ABL versus PCoA ordinal regression results, (C) heat map of enriched pathways in the L_N165 group, average abundance > 0.1%, and (D) heat map of enriched pathways in the L_K12 group, average abundance > 0.1%.

A Spearman correlation heatmap analysis at the genus level was performed (Figure 5A). At the genus level, the ABL values were positively correlated with *Enterococcus*, *Coriobacteriaceae_UCG‐002*, *Bifidobacterium, Peptostreptococcus*, *Bilophila*, and Lactobacillus. The ABL values were negatively correlated with *Globicatella*, *Neisseria*, *Rodentibacter*, *unclassified_f__Pasteurellaceae*, and *Rothia*. TNF‐α and IL‐6 levels were both negatively correlated with *Rhodanobacter*, *Mesotoga*, *Pseudonocardia*, *Paludibacter*, and *Thiobacillus*. To further explore the degree of correlation between indicators of pathological periodontitis and oral flora, the correlations between oral β‐diversity and ABL. We found that there is a high correlation between bacterial abundance and periodontitis pathological indicators. The results of the PCoA were subjected to ordinal regression analysis, and the results are shown in Figure 5B. As the value of ABL increased, the value of the PC1 axis PCoA decreased, and the two were negatively correlated and significantly different (*p* < 0.05).

**FIGURE 6:**
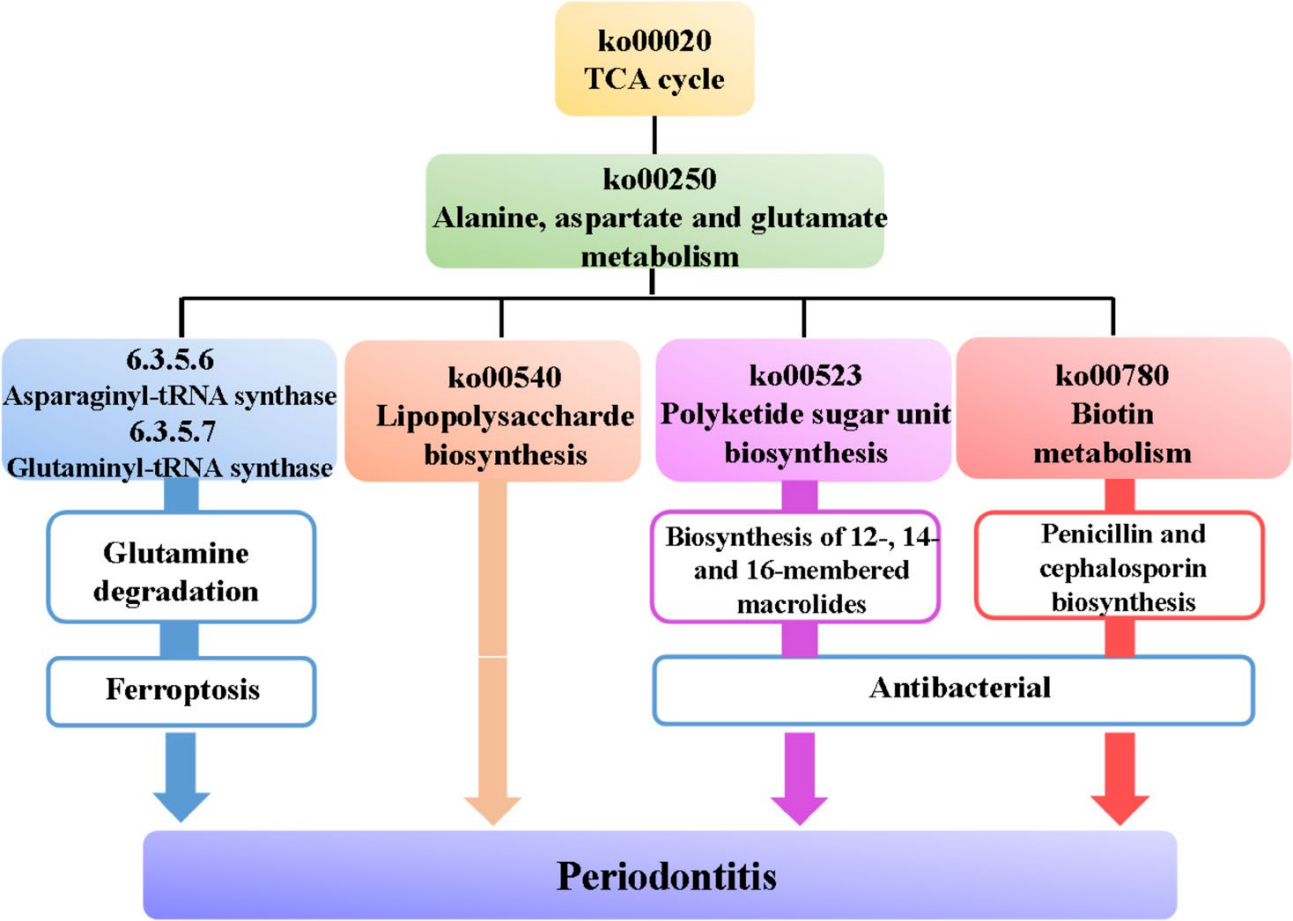
Possible action pathway of *L. zeae* N165 in regulating oral flora of periodontitis rats.

To explore the function of the dominant groups in the strains, high‐throughput sequencing results were used for PICRUSt function prediction, and the pathway enrichment heatmap of Pathway 3 is shown in Figure 5C,D. There was a similarity of the enriched pathways in the N165 and K12 groups; however, there was no average abundance > 0.1% in the pathways in group 77. The enriched pathways from the KEGG database analysis indicated significant differences between the screened model group and the CK group, and there was a significant regulation of the pathways of action in the subject group. We speculated that the possible enriched pathways were those shown in Figure 6, and that the N165 group was involved in the tricarboxylic acid (TCA) cycle as well as the amino acid metabolism pathway, suggesting that it could alleviate periodontitis via lipopolysaccharide, ketoacylglycosan unit biosynthesis, or biotin metabolism.

### 3.4 Effects of Strains on the Intestinal Flora of Rats

**FIGURE 7:**
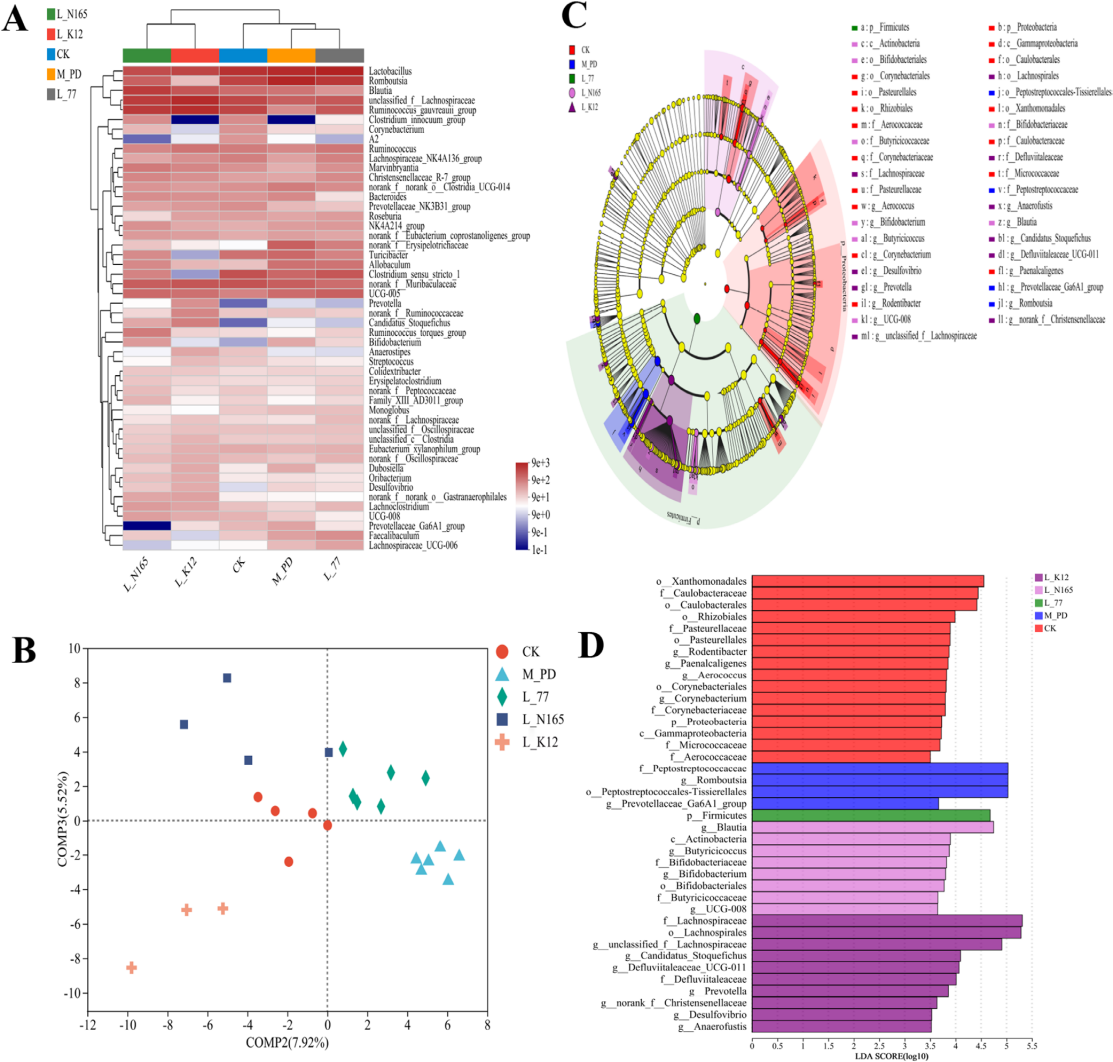
Effect of strain on intestinal flora in rats. (A) Heat map of genus level community distribution, (B) Analysis results of PLS‐DA, (C) LEfSe multilevel species hierarchical tree, (D) LDA discrimination results. LDA threshold 3.5.

The effect of the strains on the intestinal flora at the genus level was further explored. As shown in Figure 7A, the intestinal community structure of the N165 and K12 groups was significantly different from that of the model group and was similar to that of the CK group, and the 77 group showed significant similarity to the model group. This suggested that *L. zeae* N165 was superior to *L. casei* 77 in maintaining a healthy intestinal community structure. To further investigate the effects of different lactic acid bacteria on the intestinal flora associated with periodontitis, a PLS‐DA analysis was performed, as shown in Figure 7B. There was a significant difference between the model group and the CK group, and the differences between the N165 group, the 77 group, and the K12 group compared to the CK group were all less than that between these groups and the model group, but the similarity between the 77 group and the model group was much higher than that between the N165 and K12 groups. The structure of the intestinal flora of group N165 and group K12 was more similar to that of group CK (Figure 7B). To further explore the key species of lactic acid bacteria involved in the intestinal flora, a LEfSe multilevel species difference analysis, as shown in Figure 7C,D. The dominant species in the model group were *f__Peptostreptococcaceae*, *g__Romboutsia*, *o__Peptostreptococcales‐ Tissierellales*, and *g__Prevotellaceae_Ga6A1_group*. The dominant species in group 77 was *p_Firmicutes*. *g__Blautia*, *c__Actinobacteria*, and *g__Butyricicoccus* were the dominant species in group N165. The dominant species in group K12 included *f__Lachnospiraceae* and *o__Lachnospirales*.

## 4 Discussion

### 4.1 Probiotic Potential of Screened Strains on Oral Health

Auto‐aggregation refers to the phenomenon in which strains form mutual agglutination between bacteria of the same species through specific recognition of surface molecules, and strains with higher auto‐aggregation ability indicate that they may have higher adhesion ability in the oral cavity (Piwat et al. 2015). Some studies have shown that co‐aggregation refers to the phenomenon of co‐aggregation of different species of bacteria in the oral cavity, and the co‐aggregation of Lactobacillus and oral pathogens in the oral cavity can effectively prevent pathogenic bacteria from combining or colonizing, thus reducing the harmful effects of pathogenic bacteria and maintaining oral health (Piwat et al. 2015). Since the alternative strains were effective against both *F. nucleatum* and *P. gingivalis* , the co‐aggregation ability of Lactobacillus with both strains of oral pathogens was determined in the present study, and the results showed that *L. zeae N165* had a high rate of self‐aggregation at 87.75%, and co‐aggregation rates with *F. nucleatum* and *P. gingivalis* of 96.78% and 88.94%, respectively.

### 4.2 Mitigating Effect on Periodontitis of Freeze‐Dried *L. zeae* N165

Based on an in vitro study, we evaluated the mitigating effect of *L. zeae* N165 freeze‐dried alive cells on periodontitis in rats. Currently, the methods for analyzing alveolar bone in animal models of periodontitis include histomorphometric measurement and two‐dimensional radiography. Traditional methods can only provide two‐dimensional or linear data. In contrast, Micro‐CT analysis can evaluate bone mass and intraosseous microstructure in a non‐destructive, rapid, and highly accurate manner (Yang et al. 2014). Alveolar bone loss is one of the most important clinical manifestations of periodontitis (Tonsekar et al. 2017). Alveolar bone resorption is the distance from the enamel bone border to the alveolar ridge and is a key indicator of the severity of periodontitis. The lower the value of alveolar bone resorption, the less severe the symptoms of periodontitis. In this study, Micro‐CT was used to observe the microstructure of alveolar bone in rats, and the results showed that *L. zeae N165* could alleviate the alveolar bone resorption caused by periodontitis, while the K12 strain did not have a significant effect. Periodontitis can stimulate the production of protein cytokines, including interleukin‐10 (IL‐10) and interleukin‐6 (IL‐6), that along with tumor necrosis factor‐alpha (TNF‐α) orchestrate the local immune response (Jain et al. 2020). TNF‐α is a pro‐inflammatory cytokine released by macrophages that plays a central role in inflammatory responses and affects cells involved in osteoclast formation, including macrophages, stromal cells, and RANKL‐expressing T‐cells, resulting in osteoclastic potential (Kitaura et al. 2022). IL‐6 is present in endothelial cells, fibroblasts, and macrophages in patients with periodontitis and is a potent stimulator of osteoclast differentiation and bone resorption by influencing the composition of the subgingival microbiota and increasing the susceptibility to colonization by periodontal pathogenic bacteria (Shao et al. 2009). We found through monitoring TNF‐α and IL‐6 that *L. zeae* N165 alleviated elevated inflammatory factors due to periodontitis.

### 4.3 Intervention on the Oral Flora of Freeze‐Dried *L. zeae* N165

By analyzing the diversity of oral and intestinal flora in rats, we found that the trends of *Proteobacteria* and *Firmicutes* at the phylum level, and *Corynebacterium* and *Rothia* at the genus level in the model and control groups were consistent with the findings of previous researchers such as previous studies (Shi et al. 2020; Galimanas et al. 2014), but the results for Actinobacteriota at the phylum level and *unclassified_f_Pasteurellaceae* and *Veillonella* at the genus level were in contrast to the trends reported in the literature. *Actinobacteriota* at the phylum level, *unclassified_f_Pasteurellaceae* at the genus level, and *Veillonella* had opposite trends to those reported in the literature (Shi et al. 2020; Jia et al. 2021; Kawamoto et al. 2021). At the genus level of the intestinal flora, we found a similar phenomenon: the model group trends for *Romboutsia* (Yip et al. 2021), *Clostridium_sensu_stricto‐1* (Yip et al. 2021), and *unclassified_f_Lachnospiraceae* (Dong et al. 2022) were in accordance with those of previous studies, while the trends for *norank_f_ Muribaculaceae*, *Uricibacter*, *Ruminococcus* (Wu, Fang, et al. 2022; Wu, Han, et al. 2022), and *Blautia* (Bao et al. 2022) were opposite to those reported in the literature. However, the trends for *L. zeae* N165 on the regulation of macroflora were similar to those of the CK group.

We further analyzed the structure of the oral and intestinal flora at the genus level by constructing a heat map and employing PCoA and PLS‐DA. Studies on oral ulcers have shown that after administering *Bifidobacterium animalis* J‐12 to animals via gavage, wound healing can be effectively promoted by regulating the intestinal microbiota. This strain can also regulate the human immune system and enhance antioxidant capacity, thereby promoting the healing of oral ulcers, DNA repair, and other processes. We found that *L. zeae* N165 could maintain a healthy oral and intestinal community structure, similar to the CK group. We analyzed the differences in the dominant species between the oral and intestinal tracts. The dominant species *f_Atopobiaceae* and *c__Coriobacteriia* in group 77 of the oral flora have been shown in the literature to be negatively correlated with periodontal health (Ai et al. 2017), the dominant species *g__Abiotrophia* in group N165 and *g__Chryseobacterium* were positively correlated with periodontal health in a study (Muñoz Navarro et al. 2022). *c__Ktedonobacteria*, although not reported in the literature to be associated with the oral cavity, has been shown to have the potential to produce antimicrobial compounds (Zheng et al. 2019). The dominant species *f__Peptostreptococcaceae and g__Romboutsia* in the model group of intestinal flora were negatively associated with periodontal inflammatory diseases (Kawamoto et al. 2021), *g_Butyricoccus* in the N165 group showed a positive association with periodontal health in the literature (Liu et al. 2021), *g_unclassified_f__Lachnospiraceae* (Dong et al. 2022) was positively associated with periodontal health, and *Defluviitaleaceae_UCG‐011* was negatively associated with periodontal health. Thus, *L. zeae* N165 may support oral health by regulating various taxa of the oral and intestinal flora to become the dominant species.

The pathological changes involved in periodontitis may be correlated with changes in oral flora. Higher levels of ABL, TNF‐α, and IL‐6 represent more severe periodontitis. While *Coriobacteriaceae_UCG‐002* was the dominant species in group 77, *Rhodanobacter* and *f_pseudonocardiaceae* were the dominant species in group N165, and *unclassified_f__Pasteurellaceae* was the dominant species in group K12. *Rodentibacter* and *Rothia* abundance in Figure 4B showed enrichment in the N165 and K12 groups. Therefore, *S. salivarius* K12 could regulate the level of alveolar bone loss by becoming the dominant species, whereas *L. zeae N165* could regulate the level of alveolar bone loss, TNF‐α, and IL‐6 by affecting the colony abundance of dominant species, similar to the previous pathological results. In addition, we found a significant correlation between the beta diversity of oral flora and ABL values.

### 4.4 Analysis of Possible Intervention Pathways

We speculated on the possible pathways of action of *L. zeae* N165 identified by KEGG function prediction for the oral versus intestinal flora. Lipopolysaccharide, also known as endotoxin, is a virulence factor for Gram‐negative bacteria that can trigger inflammation, and oral dysbiosis in patients with periodontitis is often the result of an oral flora enriched with Gram‐negative species. Lipopolysaccharide plays a major role in the pathogenesis of periodontitis (Pussinen et al. 2022). In the present study, we found that *L. zeae* N165 had a significantly similar trend of enrichment in the pathway of lipopolysaccharide levels as the CK group. The presence of *Pseudomonas aeruginosa* is closely associated with periodontitis, and lipopolysaccharides may also modulate bacterial pathogenesis through their specific molecular features. It has been shown that *P. aeruginosa* ‐induced hyperinflammation can be alleviated by inhibiting lipopolysaccharide‐mediated signaling pathways (Qin et al. 2022), and macrolide antibiotics may also inhibit *P. aeruginosa* (da Cruz Nizer et al. 2021). These phenomena were also observed in the present study.

In addition, since endotoxemia is associated with an increased risk of cardiometabolic disorders (e.g., atherosclerosis), lipopolysaccharides can be considered as a molecular link between the periodontal microbiota and cardiometabolic disorders, and this association was found during the present study (Liu et al. 2021). Periodontitis is primarily treated by inhibiting the cell wall, proteins, or DNA synthesis of bacteria, where common cell wall inhibitors are penicillin and cephalosporin, and protein inhibitors include macrolide antibiotics (Howard et al. 2021). All of the above were found to be associated in the pathways identified in this study and are presumed to be possible pathways of action. In this study, *L. zeae* N165 and *S. salivarius* K12 were significantly enriched in the pathways of glutamine hydrolysis‐related glutamyl amino‐tRNA synthases and asparaginyl‐tRNA synthases that are potentially associated with the development of periodontitis. Ferroptosis was associated with periodontitis through oxidative stress ROS and NF‐κB‐related pathways, and there are two pathways of iron death: exogenous transporter protein‐dependent pathways (e.g., reduced and increased iron uptake by cysteine or glutamine uptake) and endogenous enzymatic pathways (Zhang et al. 2022). It has been hypothesized that *L. zeae* N165 and *S. salivarius* K12 can significantly enrich glutamyl amino‐tRNA synthetase and asparaginyl‐tRNA synthetase associated with glutamine hydrolysis and thus reduce glutamine degradation to alleviate iron death, thereby decreasing the likelihood of periodontitis triggered by the oxidative stress ROS and NF‐κB‐related pathways.

In light of the above, we hypothesized that *L. zeae* N165 may prevent the development of periodontitis through four pathways: modulation of lipopolysaccharide levels affecting Gram‐negative bacteria to produce bacterial inhibition, by producing organic acids to lower the local pH value, it reduces the direct damaging effect of LPS on periodontal tissue cells and protects the functions of gingival epithelial cells and periodontal ligament cells (Jiao et al. 2014), biosynthesis of polyketide glycan units to produce macrolide antibiotics to inhibit bacteria; this can synthesize a variety of biologically active polyketide molecules, which can inhibit the production of pro‐inflammatory cytokines, regulate the host immune response, reduce the intensity of inflammatory reactions, promote the proliferation and differentiation of gingival epithelial cells and periodontal ligament cells, and accelerate tissue repair and regeneration (Chen et al. 2024), metabolism of biotin to produce antibiotics that inhibit bacteria; it promotes the growth and proliferation of beneficial bacteria such as lactobacilli, improves microecological balance, regulates the host immune response, reduces the intensity of inflammatory reactions, and facilitates tissue repair, and reduction of the degradation of glutamine to prevent iron death; it reduces the number of bacterial communities with strong glutamine‐degrading ability and competes with periodontal pathogens for glutamine substrates to lower their availability, thereby reducing the production of harmful metabolites, a pathway that has not been previously reported.

## 5 Conclusion

In this study, an oral probiotic strain, *L.zeae* N165, was screened in vitro and evaluated the effectiveness of freeze‐dried cells against periodontitis by alleviating alveolar bone loss due to periodontitis, elevation of TNF‐α and IL‐6, maintaining a healthy oral and intestinal community structure, and regulating the dominant bacterial species in vivo. The prediction of functions by KEGG analysis of oral flora revealed that *L. zeae* N165 may alleviate periodontitis through four pathways: reduction of glutamine degradation, modulation of lipopolysaccharide levels, biosynthesis of polyketide glycan units, and biotin metabolism.

## Author Contributions

**Hongxing Zhang:** writing – review and editing, investigation, data curation. **Yuanhong Xie:** writing – review and editing, funding acquisition. **Xiaodong Song:** writing – review and editing, writing – original draft, funding acquisition. **Junhua Jin:** writing – original draft, writing – review and editing, validation, supervision, project administration, resources, investigation, methodology, funding acquisition, formal analysis, data curation, conceptualization. **Zixin Kang:** methodology, data curation, resources, project administration, validation, investigation, writing – original draft, writing – review and editing. **Qimeng Liang:** writing – review and editing, writing – original draft, validation, resources, project administration, methodology, investigation, data curation.

## Funding

The authors have nothing to report.

## Disclosure

Declaration of generative AI and AI‐assisted technologies in the writing process: During the preparation of this work, the author(s) did not use any AI and AI‐assisted technologies.

## Ethics Statement

The animal experiments were performed in accordance with the recommendations of the Animal Management Regulations, Ministry of Science and Technology of the People's Republic of China. The protocol was approved by the Ethical Committee of the Experimental Animal Care of Beijing University of Agriculture (Approval No. 812401096).

## Conflicts of Interest

The authors declare no conflicts of interest.

## Data Availability

The data that support the findings of this study are available from the corresponding author upon reasonable request.
